# Influence of the Shear Cap Size and Stiffness on the Distribution of Shear Forces in Flat Slabs

**DOI:** 10.3390/ma15010188

**Published:** 2021-12-27

**Authors:** Maciej Grabski, Andrzej Ambroziak

**Affiliations:** 1 Faculty of Civil and Environmental Engineering, Gdansk University of Technology, 11/12 Gabriela Narutowicza Street, 80-233 Gdansk, Poland; 2 Maciej Grabski Engineering, 94B/1 Leszczynowa Street, 80-175 Gdansk, Poland; grabski@gengineering.pl

**Keywords:** reinforced concrete, slab–column connections, punching shear, shear cap

## Abstract

The scope of this paper is to investigate analytically and numerically the influence of shear cap size and stiffness on the distribution of shear forces in flat slabs in a slab–column-connections-reinforced concrete structure. The effect of support (shear cap) stiffness on the calculation of the length of the shear control perimeter according to the available methods is presented. Based on the analysis, the authors indicate in what range of support stiffness the corner concentrations become important in the calculation of the punching resistance. For shear caps with high flexibility ($\alpha_{1}$ ≤ 0.5), the concentration of internal forces in the corners does not occur. The authors compare the numerical results obtained from the calculation methods and indicate the correlations, which can be useful guidance for structural designers. In the case of large shear caps, the simplified MC2010 method gives a significantly lower value of the effective control perimeter length compared to more accurate methods. This paper is intended to provide scientists, civil engineers, and designers with guidelines on which factors influence punching shear load capacity of the slab–column connections with shear caps.

## 1. Introduction

**Figure 1:**
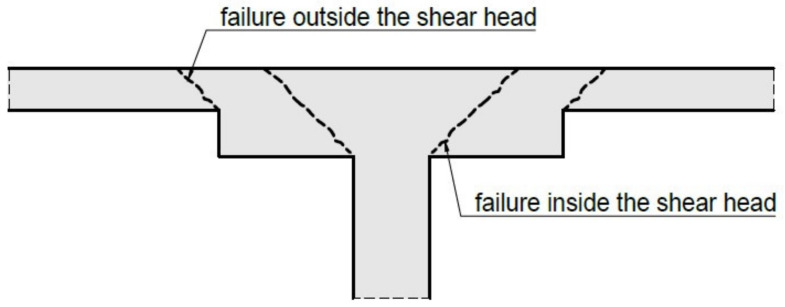
A column topped with a shear cap showing potential failure regions.

One of the key issues in the structural design of slab–column structures is a proper determination of the stress state of a nearby slab with a column connection. In the first constructions of this type, this zone was intuitively solved by widening the column near the plate (column capital) or thickening the slab on the support (shear caps). As research on punching shear has progressed, extending the area of connection between the slab and the column has been reduced for design reasons. At present, the shear caps are still widely used mainly for two reasons. Firstly, they increase the punching resistance of the slab–column connection (especially when the transverse reinforcement does not provide the required load capacity). Secondly, they increase the stiffness of the slab, which positively affects its deformation state. According to design standards, the design process for a slab to column topped with a cap connection requires verification of the punching shear resistance in the cap zone and the slab area outside the cap (see Figure 1). When considering the punching situation outside the cap zone, it is usually dealing with the case of a large-size flexible support of a square/rectangular shape. Designing the improper size of the shear caps or its wrong thickness may lead to punching shear failure of a slab–column connection and, in consequence, to a catastrophe failure of the reinforced concrete structure, e.g., [1] or [2]. Therefore, there is a need to extend knowledge of this phenomenon and raise the awareness of structural designers that the punching shear phenomenon cannot be omitted in the design process of reinforced concrete structures, especially slab–column connections.

**Figure 2:**
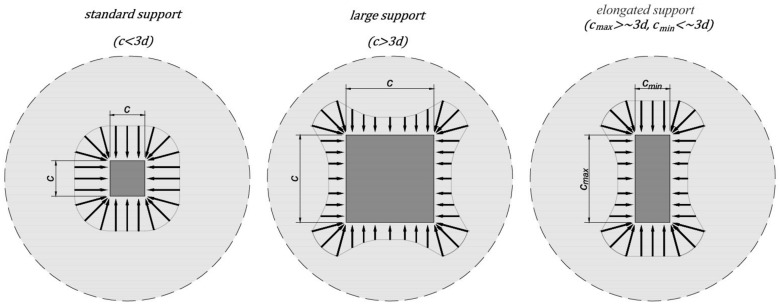
Distribution of shear forces in the vicinity of internal support as a function of its size and shape.

Slab–column connections are still a subject of much experimental research. Bonić et al. [3] investigated the punching shear capacity of reinforced concrete column footings, accounting for the soil–structure interaction effect. Urban et al. [4] presented results of an experimental test of thickset reinforced concrete slabs in punching. Schmidt et al. [5] investigated concrete and steel contributions in shear-reinforced column bases with systematically varying shear reinforcement ratios. Sahoo and Singh [6] experimentally investigated the punching shear behavior of balanced recycled-aggregate concrete-scaled slab–column specimens. Gołdyn [7] studied the effect of load level of corner columns on punching shear resistance of flat slabs. Urban et al. [8] performed experimental investigations on punching shear of lightweight aggregate concrete flat slabs. Yooprasertchai et al. [9] performed remediation of punching shear failure using glass fiber-reinforced polymer rods. Chen and Chen [10] performed the structural behavior and punching shear strength of the concrete slab–column connections strengthened with carbon-fiber-reinforced polymer laminates. In addition to experimental investigations, many theoretical studies and numerical analyses have been performed on punching shear modeling and prediction of slab–column connection behavior. Díaz et al. [11] studied numerically the punching shear resistance of unbonded post-tensioned slabs without shear reinforcement. Ricker et al. [12] gave a comprehensive review of state-of-the-art reliability techniques where the safety level of design provisions for punching shear resistance without shear reinforcement was investigated. Alotaibi et al. [13] described the prediction of punching shear capacity for fiber-reinforced concrete slabs using neuro-nomographs constructed by machine learning. Lewiński and Więch [14] performed a numerical analysis and show results for the punching shear failure of reinforced slabs. Wosatko et al. [15] described the application of damage–plasticity models in finite element analysis of punching shear. Pacheco et al. [16] gave design guidelines for the shear design of recycled-aggregate concrete elements with and without shear reinforcement. Mashrei and Mahdi [17] described an adaptive neurofuzzy inference model to predict the punching shear strength of flat concrete slabs. Koppitz et al. [18] analyzed and classified over 40 analytical punching shear models. The punching shear failure sparks a vital interest in the community of engineers, designers, and scientists to be taken into consideration, as the subject of different investigations to describe their complex mechanism of punching shear failure and behavior of slab–column connections. Scientific research (e.g., [19,20]) has indicated that for large or elongated supports the shear force distribution near the support is uneven despite symmetrical loading (Figure 2). In the case of internal columns loaded symmetrically, the unevenness of the shear forces may also be caused by the geometry of the floor slab [21,22] or the distribution of longitudinal reinforcement [23]. Many experimental data have provided evidence to confirm the impact of a nonuniform distribution of shear forces on the reduction of punching resistance [24,25,26]. At the same time, researchers have shown that advanced computational methods and analysis can lead to a better understanding of the punching shear phenomenon [27,28,29,30,31].

Over the years, standard regulations have differed from each other on some key points for the punching shear calculations. For example, until the present day the American code ACI 318 [32] protocol does not take into account the influence of the amount of longitudinal reinforcement of the slab on the punching shear capacity. On the other hand, the European EC2 [33] and international MC2010 [34] standards take into account the degree of reinforcement as one of the parameters determining the shear punching resistance of the slab–column connection.

**Figure 3:**
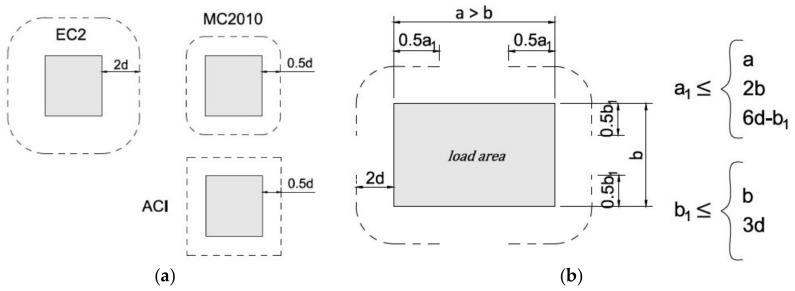

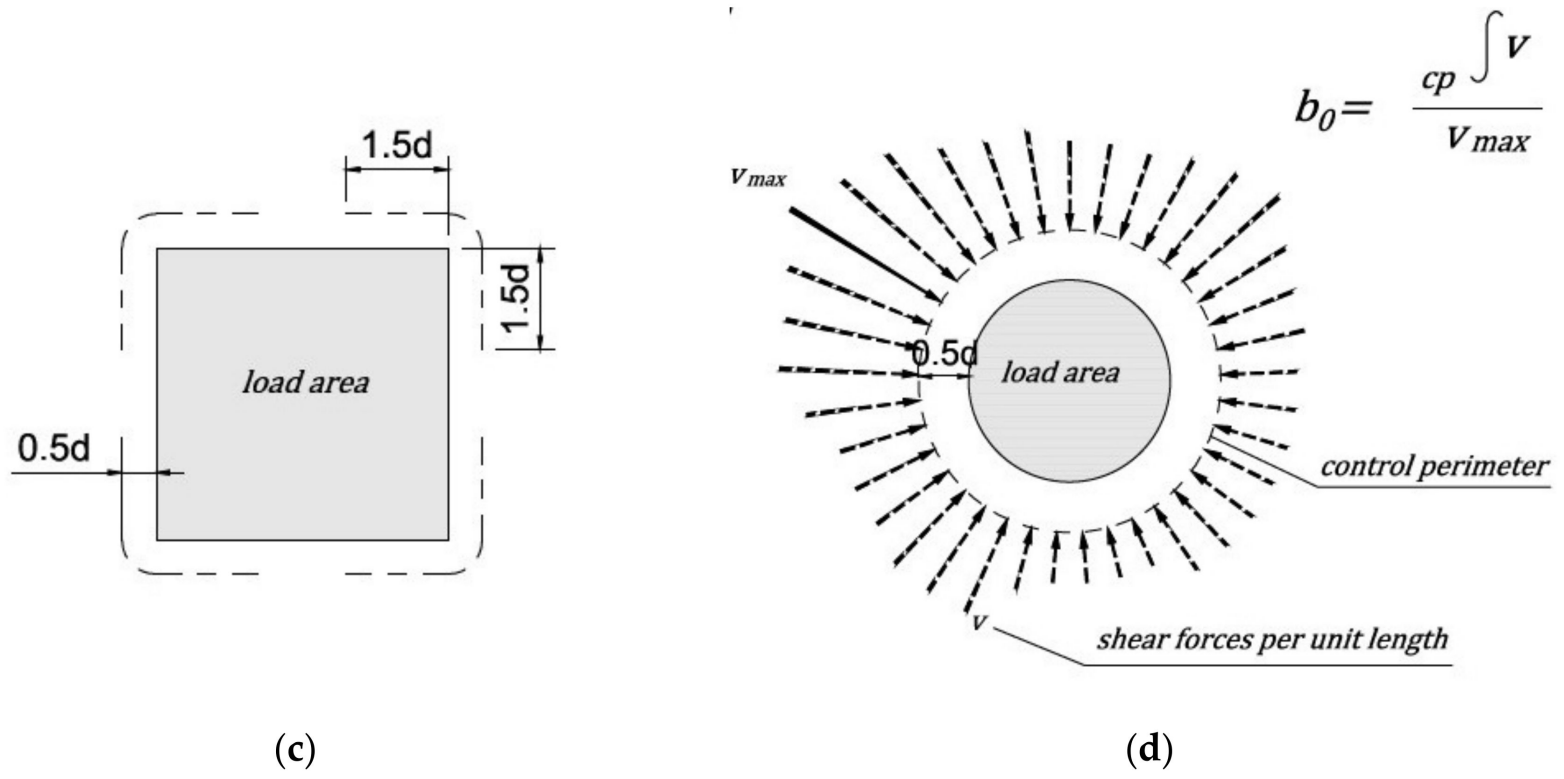
Control perimeter for punching shear according to different design methods: (**a**) basic control perimeter of ACI, EC2, MC2010; (**b**) reduction of control perimeter according to EC2-DIN; (**c**) reduction of control perimeter according to the MC2010-simplified method; (**d**) reduction of control perimeter according to the MC2010-general method.

Despite these differences, all code methods are based on the control perimeter concept. This concept assumes a certain section length (the length of the effective control perimeter ($b_{0}$) multiplied by the effective height of the plate (*d*)) based on which the punching shear capacity conditions are checked. By multiplying the area of this cross-section by the value of allowable shear stresses, the value of the permissible shear force acting on the connection is obtained. The locations of the primary control perimeter depending on the selected method are shown in Figure 3. The concept of the control perimeter is helpful for design purposes because it simplifies the standard procedures even though it does not always reflect the actual behavior of the connection. The control perimeter is therefore a key parameter of the standard methods of calculating the punching shear resistance.

In the EC2 standard [33], a nonuniform distribution of shear forces is accounted for by increasing the acting shear force by a factor β>1.0, which is a function of the moment transfer between the slab and the column in slab–column connections. The standard makes no additional recommendations as to elongated or large load areas, which has raised some concerns among researchers [19,26]. Some European countries have added restrictions on the punching for large supports in national annexes; for example, EC2-DIN [35] introduces a control perimeter length limit, as shown in Figure 3b.

The ACI code [32] does not reduce the control perimeter. The effect considered is taken into account by reducing the allowable transverse stresses depending on the size and shape of the support. The first reduction factor is decisive for supports with an elongated shape, and designated in this paper as:

(1) $$k_{e,ACI}=\left(2+\frac{4}{\beta }\right),$$

where $\beta =\frac{c_{1}}{c_{2}}>1$ and $c_{1},\;c_{2}$ are support dimensions.

The second factor, which is decisive for a large support, is described as:

(2) $$k_{e,ACI}=\left(2+\frac{\alpha_{s}\cdot d}{b_{0}}\right),$$

where α_s_ is a factor depending on the position of the column (40 internal columns). The MC2010 standard [34] introduces a simplified method that consists of reducing the control perimeter according to Figure 3c or the general method, which explicitly accounts for all effects by reducing the control perimeter to an effective control perimeter [22]:

(3) $$b_{0}=\frac{V}{v_{max}},$$

where *b*_0_ is the length of the control perimeter, *V* is the punching force, and *v_max_* is the maximum value of the shear force per unit length along the control perimeter (Figure 3d). Equation (3) assumes no redistribution of internal forces. In fact, the redistribution of internal forces occurs due to the nonlinear behavior of reinforced concrete structures [21]. This can be a rough assumption, especially in the case of large or elongated support columns. Setiawan et al. [36] proposed a modification of Equation (3) in the form:

(4) $$b_{0,\mathrm{Set}}=\frac{V}{v_{3d\;,av}},$$

where $v_{3d,\;av}$ is the average stress occurring in the part of the control perimeter reduced to the corners and the straight sections with a length 3*d* (Figure 3c).

Punching shear is one of the main failure mechanisms in slab–column connections. Despite the research that has been conducted, the calculation methods used in the codes differently take into account the effect of the nonuniform distribution of shear forces near the support face. Almost all research concerns flat plates without shear caps. It seems advisable to analyze the available methods to account for the nonuniform distribution of shear forces in the control perimeter in punching resistance calculations for a situation of the shear cap enhancement. In what range of cap stiffness does the concentration of internal forces at their corners occur, and how does it reduce the cap punching resistance through the floor?

This research presents a unique analytical and numerical analysis of the shear cap size and stiffness influence on the distribution of shear forces in flat slabs in slab–column-connections for reinforced concrete structures. The effect of support (shear cap) stiffness on the calculation of the length of the shear control perimeter according to the available methods is presented. Based on the analysis, the authors indicate in which range of support stiffness the corner concentrations become important in the calculation of the punching resistance. The authors compare the results obtained from the calculation methods considered and indicate the correlations, which can be useful guidance for designers of structures. This paper is intended to provide scientists, civil engineers, and designers with guidelines on which factors influence punching shear load capacity of slab–column connections with shear caps.

## 2. Numerical Study

### 2.1. Shear Flow in the Slabs

**Figure 4:**
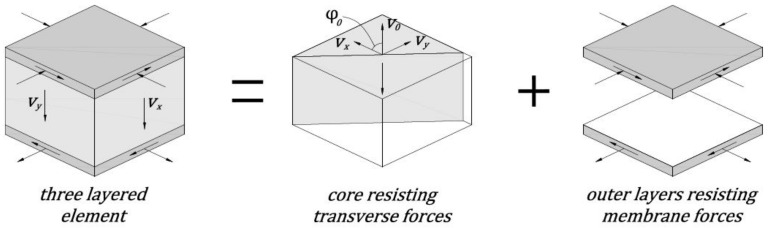
The concept of slab elements divided into three layers; see [40] for details.

Shear field analysis is used to determine the flow of shear forces in reinforced concrete slabs [21,22,37]. This analysis method is useful in the case of unconventional slab elements in which the shear forces are distributed unevenly in the analyzed control perimeter. In particular, this analysis method can be used to determine the length of the control perimeter according to the Equations (3) and (4). The shear fields in the reinforced concrete slab are the vector fields represented at each point by the direction $\left(\phi_{0}\right)$ and the magnitude $\left(v_{0}\right)$ of the main shear force [38]. The physical meaning of the parameters $\phi_{0}$ and $v_{0}$ for reinforced concrete structures can be explained using the layered model, e.g., [39]. According to the layered model, the reinforced concrete slab is divided into three layers (Figure 4). The two outer layers are responsible for the transfer of membrane forces in the plane of the concrete slab. These forces correspond to the compressive stresses in the concrete and the tensile stresses in the reinforcing steel, which are caused by the bending and torsional moments. The middle layer (the core of the reinforced concrete slab) carries only the shear stresses. The shear forces per unit length $\left(v_{x},\;v_{y}\right)$ acting in the cross-section of the element are in equilibrium with the shear forces generated in the plane of the plate. These forces are responsible for the increase in membrane forces in the slab [22]. The shear forces acting in the concrete slab plane as the resultant give the vector of the main shear force at a given point $\left(v_{0}\right)$. The direction of this vector is described by angle $\phi_{0}$. The value of the main shear force and its direction can be calculated as:

(5) $$\begin{array}{cc} v_{0}=\sqrt{v_{x}^{2}+v_{y}^{2}}, & \phi_{0}=\arctan\left(\frac{v_{y}}{v_{x}}\right) \end{array}.$$

**Figure 5:**
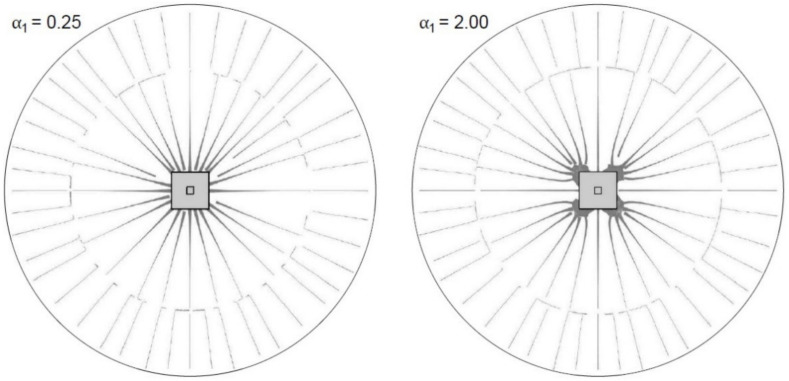
Examples of shear force flow maps obtained from the analysis.

The shear force flow maps can be obtained from finite element analysis with a linearly elastic concrete material model. In order to obtain results of internal forces similar to nonlinear calculations, coefficients reducing the stiffness of the element should be used [41]. From finite element calculations, the shear forces per unit length in the slab are obtained $\left(v_{x},\;v_{y}\right)$. After calculating the value $\phi_{0}$ and $v_{0}$, the results are presented in the form of a flow map of the main shear forces, the directions of which are defined by the angle $\phi_{0}$. The thickness of the flow line is proportional to the magnitude of the force at a given point $v_{0}$. An example of a shear force flow map is shown in Figure 5.

### 2.2. Influence of the Stiffness Modifier on the Distribution of Shear Force in FEM Calculation

**Figure 6:**
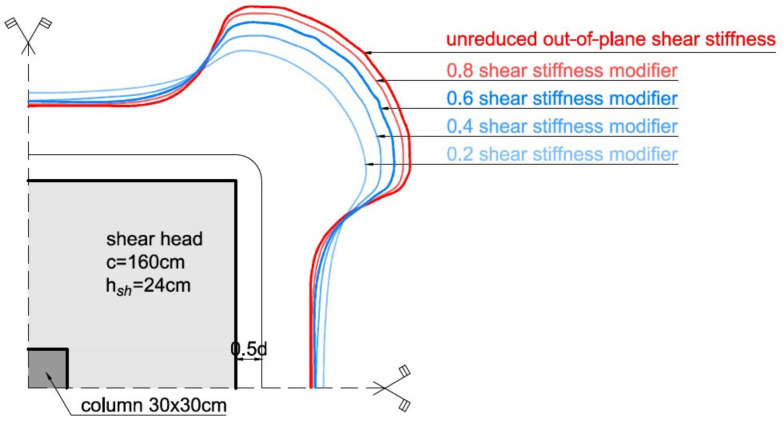
Influence of slab shear stiffness reduction to shear forces around the control perimeter at 0.5*d* from the shear cap face.

The adopted modeling method significantly influences the obtained distribution of internal forces in the slab [28,29,30,42,43]. Shu et al. [44] obtained a very good agreement of experimental results using the 3D nonlinear finite element analysis with continuum element calculations. Setiawan et al. [36] indicated that in the case of linear elastic calculations, an out-of-plane shear stiffness modifier between 0.2 to 0.4 can be used to obtain a similar distribution of shear forces in the slab for the failure stage to the distribution obtained in nonlinear calculations. To demonstrate the effect of stiffness reduction on the distribution of shear forces near the support, example calculations were performed. Figure 6 shows a plot of the shear forces in the control perimeter located at a distance of 0.5*d* from the shear cap face, depending on the reduction factor used in the calculations. The results of the calculations are presented in Table 1, where *k*_red_ is an out-of-plane shear stiffness modifier.

Application of stiffness modifiers allows for obtaining internal forces while taking into account their redistribution. For a factor equal to 0.2, the maximum shear force in the control perimeter decreases by about 30%. The averaged shear force from the part of the circular control perimeter extended by a distance of 1.5*d* on each side of the corner decreases by about 10%. Additionally, it can be seen that regardless of the coefficient modifying the plate shear stiffness, the averaged shear force $v_{av,3d}$ takes the value of about $0.7\cdot v_{max}$. The calculations presented show that assuming the averaged shear force $v_{av,3d}$ for the calculation of the length of the control perimeter (Equation (4)) gives a similar result as reducing the shear stiffness of the floor slab by a factor of 0.2–0.4.

For practical use, it is recommended to ignore the influence of the coefficients reducing stiffness [36], as further work on the issue under consideration is required. In the present analysis, the calculations were carried out without the coefficients modifying the stiffness of the element.

### 2.3. Performed Analysis

**Figure 7:**
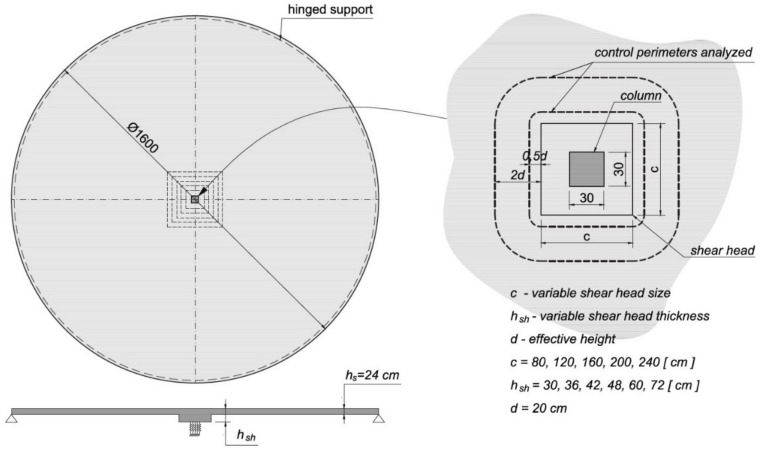
The geometry of the analyzed structural system—the influence of the dimensions and height of the shear cap.

**Figure 8:**
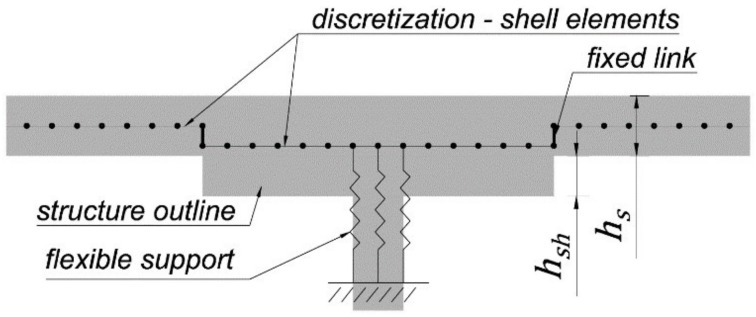
The method of modeling the slab–shear-cap connection.

The symmetrically loaded slab–column connection containing a shear cap is investigated (Figure 7). To obtain complete symmetry of the load and symmetry of forces in the slab, a plate in the shape of a circle 16 m in diameter is considered. The plate is free supported along its circumference and pointwise supported (on a column with a shear cap) in its center. The concrete slab thickness is equal to 24 cm with an effective height *d* equal to 20 cm. Within the shear cap area, a thickened surface was added, which is placed eccentrically with respect to the slab, so that the upper surfaces of both slabs are in the same level (see Figure 8). The relative thickness parameter ($\alpha_{1}=h_{sh}/h_{s}$) is introduced as the ratio of the cap height ($h_{sh}$) to the slab height ($h_{s}$). The variables of the numerical analysis are the dimensions of the shear cap and its thickness. Five cases of the shear cap size are considered: 80, 120, 160, 200, and 240 cm. Six cases of shear cap thickness are examined for each dimension: 30, 36, 42, 48, 60 and 72 cm. The thickness of the concrete slab is 24 cm; thus, the parameters of the relative height of the cap $\alpha_{1}$ for the individual thicknesses are 0.25, 0.5, 0.75, 1.0, 1.5, and 2.0. The size of the column is 30 cm by 30 cm. The concrete strength class C30/37 is used as the material for the slab–column structure. The uniform load equal to 16 kPa (6 kPa for the dead weight and an additional load of 10 kPa) is applied to the construction. The shell finite element analysis is performed in the RFEM program of Dlubal Software. The program has the ability to present the main shear forces in the cross-section of the control perimeter or any other cross-section modeled by the user. The generation of the shear flow maps is performed in a MATLAB procedure. The numerical results are obtained in the linear elastic analysis by modeling the slab as a shell element using finite elements of the MITC-type (mixed interpolation of tensorial components). The mesh independence study of the slab–column-connection finite element model is carried out to ensure that the results of an analysis are not affected by changing the size of the mesh. The column support is modeled as flexible surface support with dimensions equal to the dimensions of the column and the stiffness calculated based on its material and geometric characteristics. This approach is capable of reflecting the column support realistically [27].

## 3. Results of Numerical Analysis

**Figure A1:**
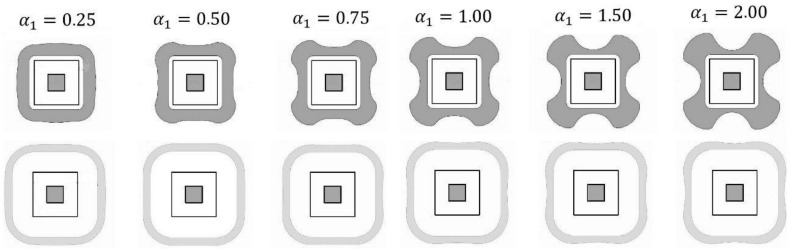
Graphs of the shear forces in the control perimeter depending on the shear cap height—80 cm shear cap width (c/d = 4).

**Figure A2:**
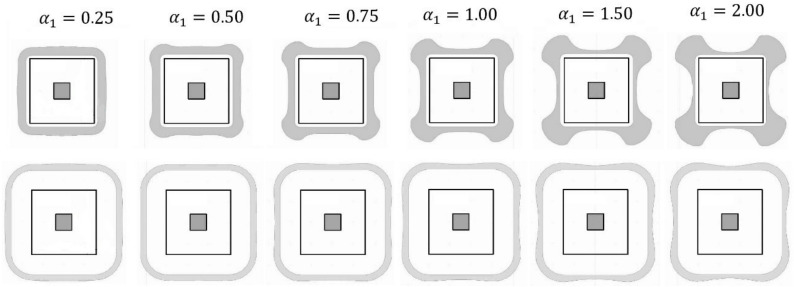
Graphs of the shear forces in the control perimeter depending on the shear cap height—120 cm shear cap width (c/d = 6).

**Figure A3:**
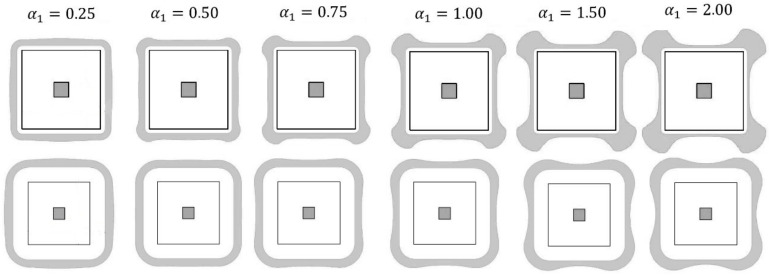
Graphs of the shear forces in the control perimeter depending on the shear cap height—160 cm shear cap width (c/d = 8).

**Figure A4:**
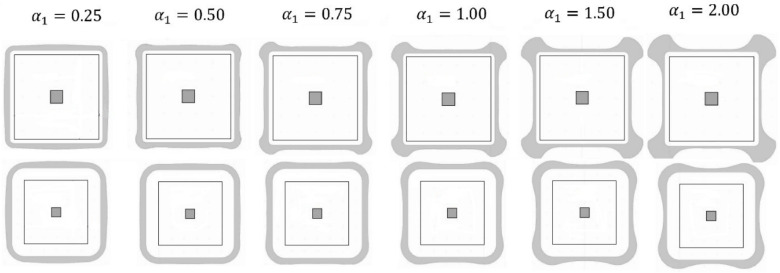
Graphs of the shear forces in the control perimeter depending on the shear cap height—200 cm shear cap width (c/d = 10).

**Figure A5:**
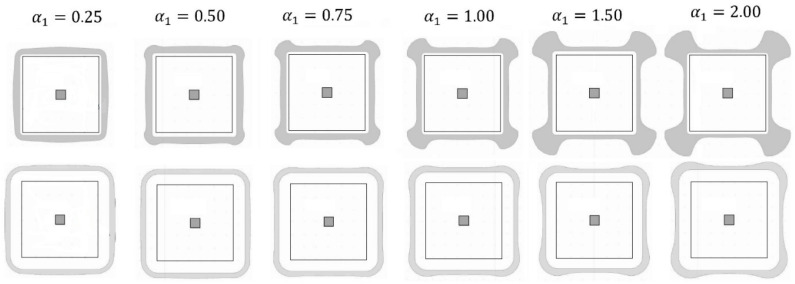
Graphs of the shear forces in the control perimeter depending on the shear cap height—240 cm shear cap width (c/d = 12).

The diagrams of the main shear forces in the control perimeters distant from the shear cap face by 0.5*d* (10 cm) and 2.0*d* (40 cm) are investigated. These graphs are shown in Figure A1, Figure A2, Figure A3, Figure A4 and Figure A5. The shaded portion represents the values of the shear force (in kN/m) at a given point in the control perimeter. Parameter $\alpha_{1}$, denoting the relative thickness of the cap, is given for each map.

The data calculated from the analysis are summarized in Appendix A, Table A1, Table A2, Table A3, Table A4, Table A5, Table A6, Table A7, Table A8, Table A9 and Table A10. The punching force (*V*) is determined by integrating the shear force diagram along the length of the control perimeter. The averaged unit shear force on the entire control perimeter is marked as $v_{av}$ $\left(v_{av}=V/b_{1}\right)$, while the maximum force in the control perimeter as $v_{max}$. Following this, the part of the total punching force carried by the reduced control perimeter $V\left(b_{0,3d}\right)/V$ is calculated. The average unit shear force at the corners of the shear cap is denoted as $v_{3d,av}\;(v_{3d,av}=V\left(b_{0,3d}\right)/b_{0,3d}$). The base length of the control perimeter is described as $b_{1}$. The length reduced to the corners with 3*d* straight interval (see Figure 3c) is marked as $b_{0,3d}$. According to Equations (3) and (4), the length of the control perimeter given by the general method of the MC2010 standard [45] ($b_{0}$) and the length of the control perimeter proposed by [46] $b_{0,\;Set}$ are determined. The last three columns of the Table A1, Table A2, Table A3, Table A4, Table A5, Table A6, Table A7, Table A8, Table A9 and Table A10 show the percentage reduction of the length of the control perimeter depending on the assumed calculation methods.

The shear force diagrams presented and the analysis of the data obtained by calculations show a clear influence of the dimensions and thickness of the shear cap on the distribution of shear forces near the support. For the control perimeter 0.5*d* away from the support face, the cap stiffness plays a significant role in the distribution of the shear forces. At the relative shear heights $\alpha_{1}$ = 0.25 and $\alpha_{1}$ = 0.50, the distributed shear forces are nearly uniform. At $\alpha_{1}$ = 0.75 and $\alpha_{1}$ = 1.00, shear force concentrations in the corners are beginning to disclose, but the contribution of the straight part of the control perimeter (one-way shear) in the transmission of the total support reaction is also visible. With a further increase in the shear cap stiffness ($\alpha_{1}\;$> 1.00), the shear forces accumulate increasingly in the corners, while reducing the values in the straight sections of the support. The larger the shear cap size, the more intensified the force concentration effect. In the circumference 2*d* away from the shear cap face, the influence of the stiffness and the dimensions of the shear cap on the distribution of shear forces can also be seen. However, it is much smaller. The forces are distributed there almost evenly with the parameters $\alpha_{1}$ < 1.5.

**Figure 9:**
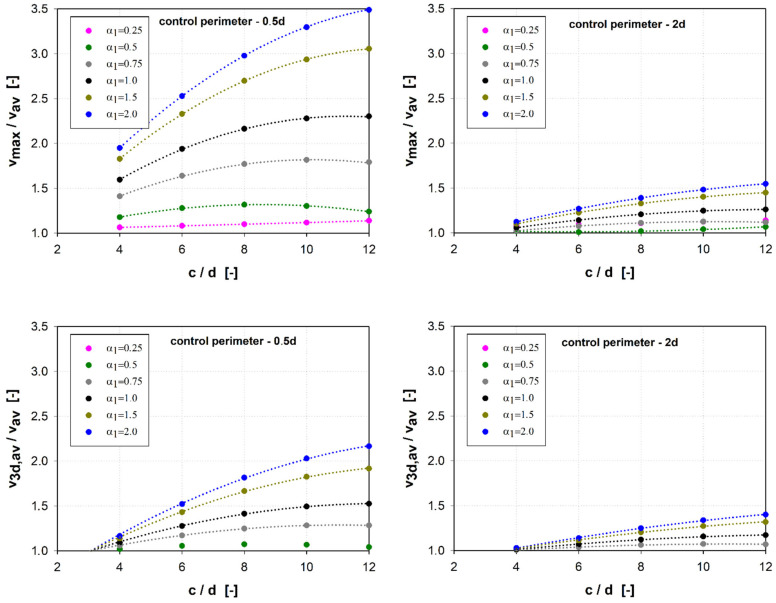
Influence of the shear cap size and its stiffness on the shear force distribution in the control perimeter located at a distance of 0.5*d* and 2*d* from the face of the shear cap.

**Figure 10:**
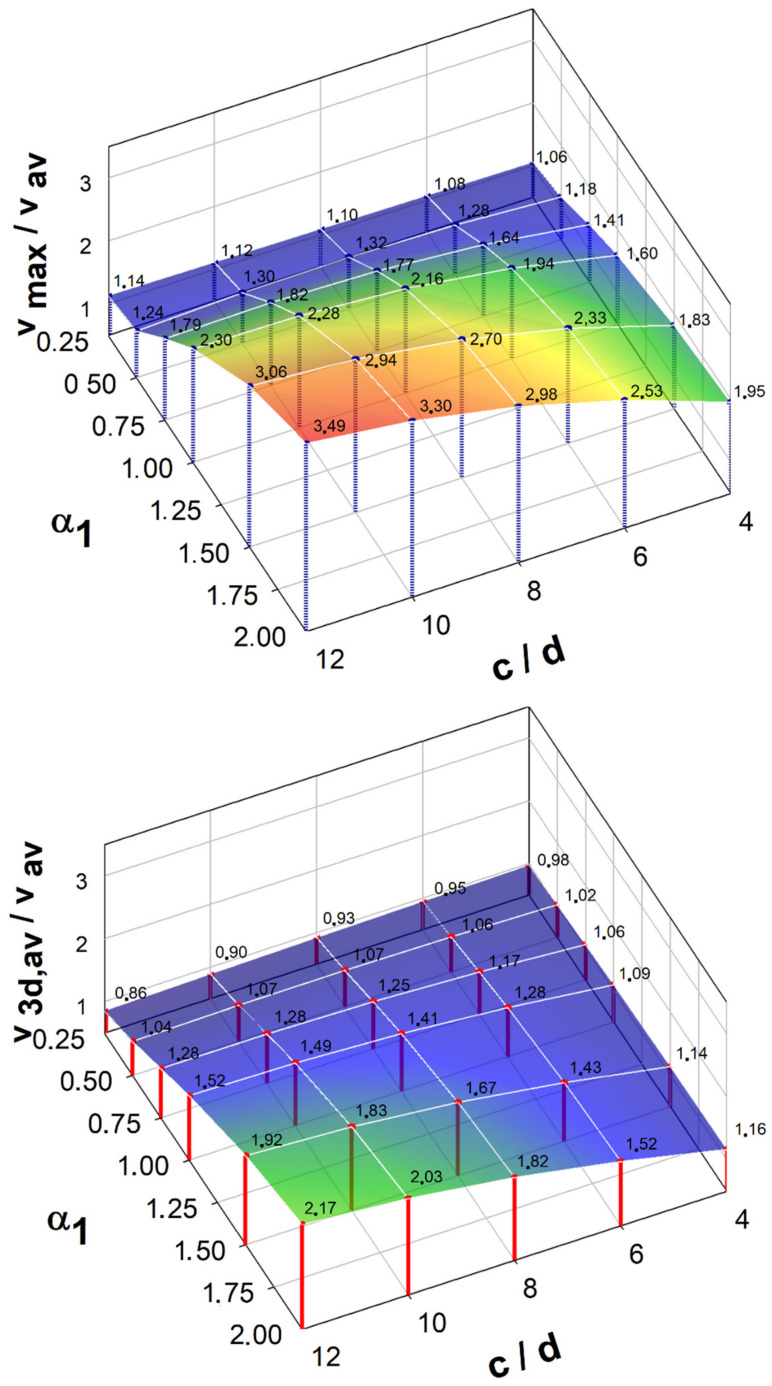
Influence of the dimensions of the shear caps and their stiffness on the distribution of shear forces in the control perimeter located at a distance of 0.5*d* from the face of the shear caps.

**Figure 11:**
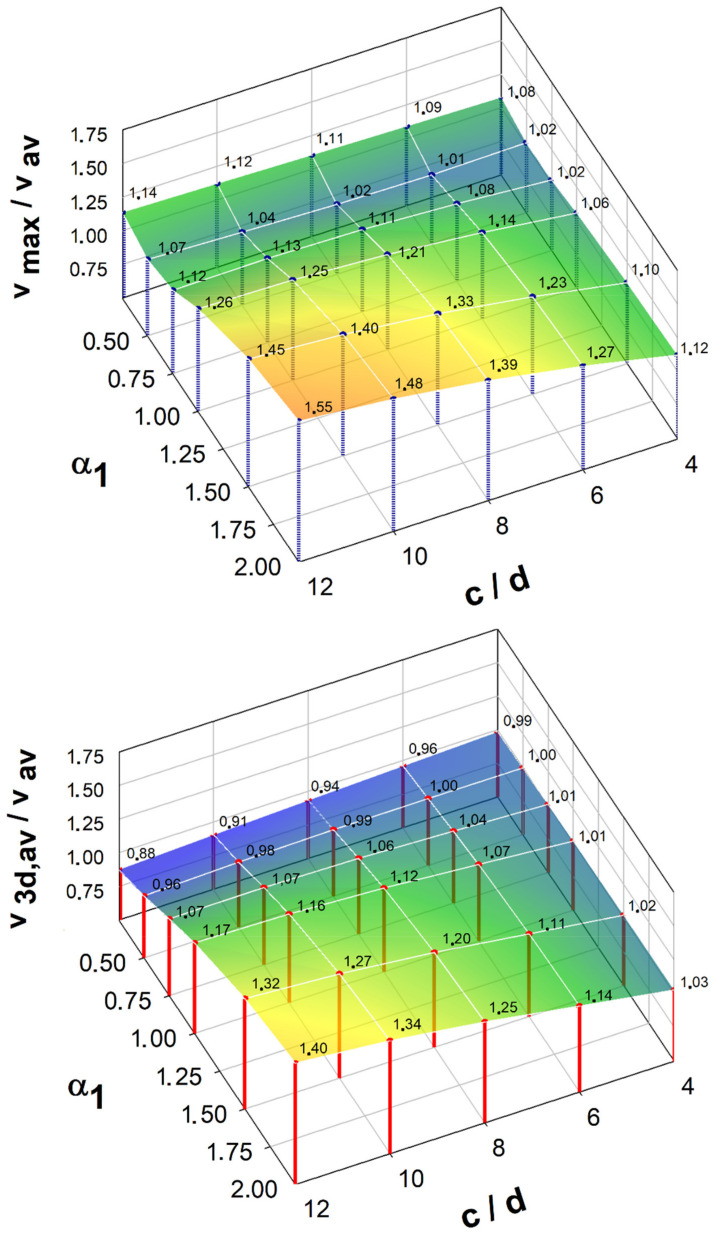
Influence of the dimensions of the shear caps and their stiffness on the distribution of shear forces in the control perimeter located at a distance of 2.0*d* from the face of the shear caps.

Taking into account the redistribution of forces in the corners proposed in [36] allows for significantly minimizing the effect of uneven force distribution in the control perimeter 0.5*d* away from the cap face (Figure 9). This effect is more noticeable for stiffer shear caps. Regardless of the cap size for caps with the relative height parameter $\alpha_{1}$ = 0.25 and $\alpha_{1}$ = 0.5, the ratio of $v_{3d,av}/v_{max}$ = ~0.85. For the remaining parameters of the relative height ($\alpha_{1}$ = 0.75–2.0) $v_{3d,av}/v_{max}$ = ~0.65. These calculations indicate a very conservative approach to the general method of calculating the shear transmitting (*b*_0_) control circuit proposed in the standard [45]. For the control perimeter located 2*d* away from the shear cap face, this effect practically does not occur. The influence of the shear cap stiffness (parameter $\alpha_{1}$) and its size (c/d) on the distribution of shear forces in the control perimeter ($v_{max}/v_{av}$ and $v_{3d,av}/v_{av}$) is shown in Figure 10 and Figure 11.

**Figure 12:**
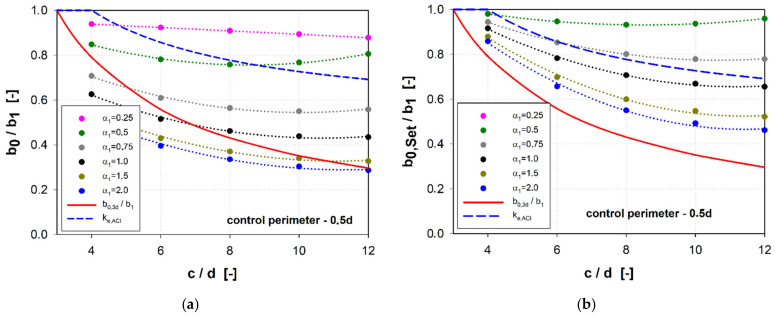
Comparison of methods for calculating the effective length of a control perimeter 0.5*d*: (**a**) reduction of the control perimeter according to the general method of MC2010 (*b*_0_/*b*_1_); (**b**) reduction of the control perimeter according to [36] (*b*_0,*Set*_/*b*_1_).

**Figure 13:**
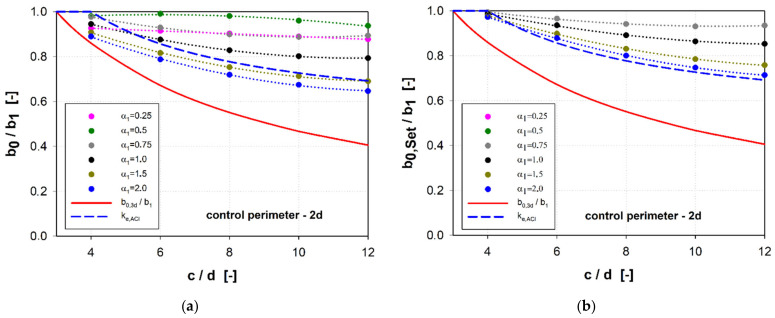
Comparison of methods for calculating the effective length of a control perimeter 2*d*: (**a**) reduction of the control perimeter according to the general method of MC2010 (*b*_0_/*b*_1_); (**b**) reduction of the control perimeter according to [36] *b*_0,*Set*_/*b*_1_.

A comparison of the calculation methods of the effective length of the control perimeter is shown in Figure 12 and Figure 13. These figures also feature the diagram of the $k_{e,ACI}$ parameter reducing the resistance of large punching shear for large load fields according to the ACI code [32]. For the control perimeter located at a distance of 0.5*d* from the shear cap face, the general method (*b*_0_) gives the results compared with the simplified method (*b*_0,3*d*_) for shear caps with the parameter $\alpha_{1}\;$≥ 1.0. For the parameter $\alpha_{1}$ < 0.75, the simplified method provides for a much greater reduction of the punching shear resistance compared to the general method. This difference is the greater the larger the size of the shear cap.

**Figure 14:**
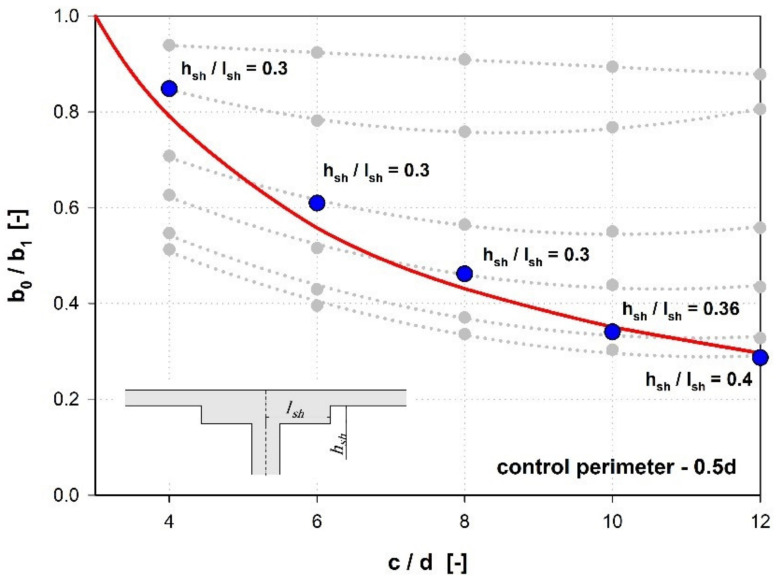
Determining the shear cap slenderness ($h_{sh}/l_{sh})$ at which the general MC2010 method for finding the effective length of control perimeter ($b_{0}$) gives comparable results to the simplified method ($b_{0,3d}$).

After entering the slenderness parameter ($h_{sh}/l_{sh}$), it can be concluded that both methods give the same results for the slenderness $h_{sh}/l_{sh}$ = ~ 0.35 (see Figure 14). Compared to the calculation method that takes into account the redistribution of the shear forces ($b_{0,set}$), regardless of the shear cap stiffness, the simplified method gives a greater reduction of the control perimeter.

The reduction of the punching shear resistance due to the support size introduced in the ACI code [32] gives results similar to the general method of the MC2010 standard [45] for slender caps ($\alpha_{1}$ < 0.5). In the case of the *b*_0,*Set*_ control perimeter, similar results were obtained for the parameter $\alpha_{1}$ = 0.75. In the case of the control perimeter located at a distance of 2*d* from the shear cap face, the results for rigid shear caps ($\alpha_{1}\;$≥ 1.5) show a reduction of the control perimeter comparable to the reduction used in the ACI code [32]. In the case of the simplified method (*b*_0,3*d*_), the reduction of the control perimeter will be much greater compared to the other methods, regardless of the redistribution of the shear forces.

## 4. Conclusions

In this paper, the influence of the shear cap dimension and stiffness on the distribution of shear forces in the control perimeters is analyzed analytically and numerically. The effect of the support (shear cap) size on the calculation of the length of the effective control perimeter according to the available methods is presented and the results obtained are compared. The main findings from the analysis follow:

- The shear force distribution in the control perimeter depends on the stiffness of the support. Ignoring shear cap stiffness in the calculation of the punching resistance in most calculation methods leads to very conservative results. However, there is a need for experimental tests to confirm the analysis of available methods.
- For shear caps with high compliance ($\alpha_{1}$ ≤ 0.5), the concentration of internal forces in the corners does not occur.
- For very large caps, the adoption of the simplified method indicated in MC2010 (*b*_0,3*d*_) gives a significantly lower value of the length of the effective control perimeter compared to more accurate methods (*b*_0_, *b*_0,*Set*_) taking into account the dimension and cap stiffness. The most conservative results compared to the more accurate methods are obtained by using a simplified method of reducing the control perimeter (*b*_0,3*d*_) for the methods developed for the control perimeter 2*d* away from the support face (EC2-DIN standard).
- Taking into account the redistribution of the shear forces in the calculations reduces the value of the extreme shear force. For caps with the relative height parameter $\alpha_{1}$ = 0.25 and $\alpha_{1}$ = 0.5, the ratio of $v_{3d,av}/v_{max}$ = ~0.85. For the remaining parameters of the relative height ($\alpha_{1}$ = 0.75–2.0) $v_{3d,av}/v_{max}$ = ~0.65. By considering the value of $v_{3d,av}$ in the calculation of the length of the effective control perimeter, the length increases by 17.6% ($\alpha_{1}$ < 0.75) and 53.8% ($\alpha_{1}\;$≥ 0.75).

The paper provides scientists, civil engineers, and designers with guidelines on the influence of shear caps size and stiffness on the distribution of shear forces in flat slabs. The result of the analysis will direct researchers to further investigate the influence of flexibility and size of the support on the distribution of internal forces in its vicinity. Without this knowledge, in the case of the slab–shear-cap connections, the proper description of the punching shear failure phenomenon is not possible. The designers of reinforced concrete structures should know which factors influence punching shear load capacity of the slab–shear-cap connections to properly design the structure and avoid the phenomenon of punching shear failure. The results obtained indicate in what range of shear cap stiffness and dimension the problem of force concentration in corners becomes relevant from the point of view of current calculation methods. As a consequence, this knowledge can lead to the design of slab–shear-cap connections that are much more economical and safe. The results obtained encourage the authors to continue research on the influence of the shear cap dimension and stiffness on the distribution of shear forces in the control perimeters.

## Figures and Tables

**Table 1:** Influence of slab out-of-plane shear stiffness reduction *k*_red_ to shear forces around the control perimeter at 0.5*d* from the shear cap face.

*k*_red_	*v_max_*		*v_av,_*_3*d*_		*v_av,_*_3*d*_ */v_max_*
	(kN/m)	(%)	(kN/m)	(%)	(%)
1	425.6	100	278.01	100	65
0.8	404.81	95	274.03	99	68
0.6	377.1	89	268.15	96	71
0.4	340.49	80	258.8	93	76
0.2	286.99	67	240.81	87	84

## Data Availability

All test results are presented in Table 1 and Table A1, Table A2, Table A3, Table A4, Table A5, Table A6, Table A7, Table A8, Table A9 and Table A10. On the request, the numerical version of the results will be provided.

## Appendix A

Results of numerical analysis of the shear force distribution in the control perimeter 0.5*d* and 2*d* with 80 to 240 cm shear cap size are presented in Table A1, Table A2, Table A3, Table A4, Table A5, Table A6, Table A7, Table A8, Table A9 and Table A10.

**Table A1:** Analysis of the shear force distribution in the control perimeter 0.5*d* with 80 cm shear cap size.

c1 = c2	c/d	α1	*V*	*V* (*b*_0.3*d*_)	*V* (*b*_0.3*d*_)*/V*	*v_av_*	*v_max_*	*v*_3*d.av*_	*v_max_/v_av_*	*v*_3*d.av*_ */v_av_*	*b*_1_	*b*_0.3*d*_	*b*_0_	*b*_0,*Set*_	*b*_0.3*d*_ */b*_1_	*b*_0_ */b*_1_	*b*_0,*Set*_ */b*_1_
(cm)	(-)	(-)	(kN)	(kN)	(%)	(kN/m)	(kN/m)	(kN/m)	(-)	(-)	(m)	(m)	(m)	(m)	(%)	(%)	(%)
80	4	0.25	1382	1075	77.8	361.2	384.4	355.0	1.06	0.98	3.83	3.03	3.60	3.89	79.1	93.9	101.7
80	4	0.5	1368	1104	80.7	357.4	421.2	364.4	1.18	1.02	3.83	3.03	3.25	3.75	79.1	84.8	98.0
80	4	0.75	1353	1133	83.7	353.6	499.3	374.2	1.41	1.06	3.83	3.03	2.71	3.62	79.1	70.8	94.5
80	4	1	1344	11,604	86.3	351.2	560.5	383.2	1.60	1.09	3.83	3.03	2.40	3.51	79.1	62.6	91.6
80	4	1.5	1338	1204	90.0	349.7	639.3	397.7	1.83	1.14	3.83	3.03	2.09	3.37	79.1	54.7	87.9
80	4	2	1341	1236	92.2	350.6	683.5	408.3	1.95	1.16	3.83	3.03	1.96	3.29	79.1	51.3	85.8

**Table A2:** Analysis of the shear force distribution in the control perimeter 2*d* with 80 cm shear cap size.

c1 = c2	c/d	α1	*V*	*V* (*b*_0.3*d*_)	*V* (*b*_0.3*d*_)*/V*	*v_av_*	*v_max_*	*v*_3*d.av*_	*v_max_/v_av_*	*v*_3*d.av*_ */v_av_*	*b*_1_	*b*_0.3*d*_	*b*_0_	*b*_0,*Set*_	*b*_0.3*d*_ */b*_1_	*b*_0_ */b*_1_	*b*_0,*Set*_ */b*_1_
(cm)	(-)	(-)	(kN)	(kN)	(%)	(kN/m)	(kN/m)	(kN/m)	(-)	(-)	(m)	(m)	(m)	(m)	(%)	(%)	(%)
80	4	0.25	1311	1113	84.9	229.5	247.7	226.5	1.08	0.99	5.71	4.91	5.29	5.79	86.0	92.6	101.3
80	4	0.5	1312	1124	85.7	229.6	234.2	228.8	1.02	1.00	5.71	4.91	5.60	5.73	86.0	98.0	100.3
80	4	0.75	1310	1133	86.5	229.4	234.8	230.7	1.02	1.01	5.71	4.91	5.58	5.68	86.0	97.7	99.4
80	4	1	1309	1141	87.1	229.2	242.5	232.2	1.06	1.01	5.71	4.91	5.40	5.64	86.0	94.5	98.7
80	4	1.5	1308	1151	88.0	229.0	252.1	234.2	1.10	1.02	5.71	4.91	5.19	5.59	86.0	90.8	97.8
80	4	2	1308	1157	88.4	228.9	257.3	235.4	1.12	1.03	5.71	4.91	5.08	5.56	86.0	89.0	97.2

**Table A3:** Analysis of the shear force distribution in the control perimeter 0.5*d* with 120 cm shear cap size.

c1 = c2	c/d	α1	*V*	*V* (*b*_0.3*d*_)	*V* (*b*_0.3*d*_)*/V*	*v_av_*	*v_max_*	*v*_3*d.av*_	*v_max_/v_av_*	*v*_3*d.av*_ */v_av_*	*b*_1_	*b*_0.3*d*_	*b*_0_	*b*_0,*Set*_	*b*_0.3*d*_ */b*_1_	*b*_0_ */b*_1_	*b*_0,*Set*_ */b*_1_
(cm)	(-)	(-)	(kN)	(kN)	(%)	(kN/m)	(kN/m)	(kN/m)	(-)	(-)	(m)	(m)	(m)	(m)	(%)	(%)	(%)
120	6	0.25	1388	739	53.3	255.9	276.9	244.2	1.08	0.95	5.43	3.03	5.01	5.69	55.8	92.4	104.8
120	6	0.5	1381	814	58.9	254.6	325.6	268.7	1.28	1.06	5.43	3.03	4.24	5.14	55.8	78.2	94.7
120	6	0.75	1368	895	65.4	252.1	413.5	295.4	1.64	1.17	5.43	3.03	3.31	4.63	55.8	61.0	85.3
120	6	1	1358	968	71.3	250.3	485.4	319.6	1.94	1.28	5.43	3.03	2.80	4.25	55.8	51.5	78.3
120	6	1.5	1355	1083	79.9	249.7	581.9	357.5	2.33	1.43	5.43	3.03	2.33	3.79	55.8	42.9	69.8
120	6	2	1368	1162	84.9	252.1	637.4	383.6	2.53	1.52	5.43	3.03	2.15	3.57	55.8	39.5	65.7

**Table A4:** Analysis of the shear force distribution in the control perimeter 2*d* with 120 cm shear cap size.

c1 = c2	c/d	α1	*V*	*V* (*b*_0.3*d*_)	*V* (*b*_0.3*d*_)*/V*	*v_av_*	*v_max_*	*v*_3*d.av*_	*v_max_/v_av_*	*v*_3*d.av*_ */v_av_*	*b*_1_	*b*_0.3*d*_	*b*_0_	*b*_0,*Set*_	*b*_0.3*d*_ */b*_1_	*b*_0_ */b*_1_	*b*_0,*Set*_ */b*_1_
(cm)	(-)	(-)	(kN)	(kN)	(%)	(kN/m)	(kN/m)	(kN/m)	(-)	(-)	(m)	(m)	(m)	(m)	(%)	(%)	(%)
120	6	0.25	1315	850	64.6	179.9	196.6	172.9	1.09	0.96	7.31	4.91	6.69	7.61	67.2	91.5	104.0
120	6	0.5	1316	882	67.0	180.0	181.6	179.4	1.01	1.00	7.31	4.91	7.25	7.34	67.2	99.1	100.3
120	6	0.75	1314	914	69.6	179.7	193.2	186.0	1.08	1.04	7.31	4.91	6.80	7.06	67.2	93.0	96.6
120	6	1	1313	942	71.8	179.6	205.0	191.8	1.14	1.07	7.31	4.91	6.41	6.85	67.2	87.6	93.6
120	6	1.5	1316	984	74.7	180.0	220.5	200.3	1.23	1.11	7.31	4.91	5.97	6.57	67.2	81.6	89.9
120	6	2	1321	1010	76.4	180.6	229.3	205.5	1.27	1.14	7.31	4.91	5.76	6.43	67.2	78.8	87.9

**Table A5:** Analysis of the shear force distribution in the control perimeter 0.5*d* with 160 cm shear cap size.

c1 = c2	c/d	α1	*V*	*V* (*b*_0.3*d*_)	*V* (*b*_0.3*d*_)*/V*	*v_av_*	*v_max_*	*v*_3*d.av*_	*v_max_/v_av_*	*v*_3*d.av*_ */v_av_*	*b*_1_	*b*_0.3*d*_	*b*_0_	*b*_0,*Set*_	*b*_0.3*d*_ */b*_1_	*b*_0_ */b*_1_	*b*_0,*Set*_ */b*_1_
(cm)	(-)	(-)	(kN)	(kN)	(%)	(kN/m)	(kN/m)	(kN/m)	(-)	(-)	(m)	(m)	(m)	(m)	(%)	(%)	(%)
160	8	0.25	1383	553	40.0	196.9	216.5	182.6	1.10	0.93	7.03	3.03	6.39	7.57	43.1	90.9	107.8
160	8	0.5	1389	642	46.2	197.7	260.6	212.1	1.32	1.07	7.03	3.03	5.33	6.55	43.1	75.9	93.2
160	8	0.75	1383	744	53.8	196.8	348.6	245.6	1.77	1.25	7.03	3.03	3.97	5.63	43.1	56.4	80.1
160	8	1	1375	838	60.9	195.6	423.3	276.6	2.16	1.41	7.03	3.03	3.25	4.97	43.1	46.2	70.7
160	8	1.5	1371	985	71.8	195.1	526.6	325.1	2.70	1.67	7.03	3.03	2.60	4.22	43.1	37.0	60.0
160	8	2	1385	1084	78.3	197.2	587.2	358.1	2.98	1.82	7.03	3.03	2.36	3.87	43.1	33.6	55.1

**Table A6:** Analysis of the shear force distribution in the control perimeter 2*d* with 160 cm shear cap size.

c1 = c2	c/d	α1	*V*	*V* (*b*_0.3*d*_)	*V* (*b*_0.3*d*_)*/V*	*v_av_*	*v_max_*	*v*_3*d.av*_	*v_max_/v_av_*	*v*_3*d.av*_ */v_av_*	*b*_1_	*b*_0.3*d*_	*b*_0_	*b*_0,*Set*_	*b*_0.3*d*_ */b*_1_	*b*_0_ */b*_1_	*b*_0,*Set*_ */b*_1_
(cm)	(-)	(-)	(kN)	(kN)	(%)	(kN/m)	(kN/m)	(kN/m)	(-)	(-)	(m)	(m)	(m)	(m)	(%)	(%)	(%)
160	8	0.25	1311	676	51.6	147.2	163.0	137.7	1.11	0.94	8.91	4.91	8.05	9.53	55.1	90.3	106.9
160	8	0.5	1315	721	54.8	147.6	150.4	146.7	1.02	0.99	8.91	4.91	8.74	8.97	55.1	98.1	100.6
160	8	0.75	1314	769	58.5	147.4	163.9	156.6	1.11	1.06	8.91	4.91	8.01	8.39	55.1	89.9	94.1
160	8	1	1315	813	61.8	147.6	178.2	165.5	1.21	1.12	8.91	4.91	7.38	7.95	55.1	82.8	89.1
160	8	1.5	1326	880	66.3	148.8	197.5	179.0	1.33	1.20	8.91	4.91	6.71	7.41	55.1	75.3	83.1
160	8	2	1338	921	68.9	150.1	208.7	187.5	1.39	1.25	8.91	4.91	6.41	7.14	55.1	71.9	80.1

**Table A7:** Analysis of the shear force distribution in the control perimeter 0.5*d* with 200 cm shear cap size.

c1 = c2	c/d	α1	*V*	*V* (*b*_0.3*d*_)	*V* (*b*_0.3*d*_)*/V*	*v_av_*	*v_max_*	*v*_3*d.av*_	*v_max_/v_av_*	*v*_3*d.av*_ */v_av_*	*b*_1_	*b*_0.3*d*_	*b*_0_	*b*_0,*Set*_	*b*_0.3*d*_ */b*_1_	*b*_0_ */b*_1_	*b*_0,*Set*_ */b*_1_
(cm)	(-)	(-)	(kN)	(kN)	(%)	(kN/m)	(kN/m)	(kN/m)	(-)	(-)	(m)	(m)	(m)	(m)	(%)	(%)	(%)
200	10	0.25	1368	431	31.5	158.6	177.4	142.2	1.12	0.90	8.63	3.03	7.71	9.62	35.1	89.4	111.5
200	10	0.5	1389	521	37.5	161.1	209.8	171.9	1.30	1.07	8.63	3.03	6.62	8.08	35.1	76.8	93.7
200	10	0.75	1393	628	45.1	161.5	293.4	207.3	1.82	1.28	8.63	3.03	4.75	6.72	35.1	55.0	77.9
200	10	1	1389	728	52.5	161.0	367.0	240.6	2.28	1.49	8.63	3.03	3.78	5.77	35.1	43.9	66.9
200	10	1.5	1385	888	64.1	160.6	471.5	293.2	2.94	1.83	8.63	3.03	2.94	4.72	35.1	34.0	54.8
200	10	2	1397	995	71.3	161.9	533.8	328.7	3.30	2.03	8.63	3.03	2.62	4.25	35.1	30.3	49.3

**Table A8:** Analysis of the shear force distribution in the control perimeter 2*d* with 200 cm shear cap size.

c1 = c2	c/d	α1	*V*	*V* (*b*_0.3*d*_)	*V* (*b*_0.3*d*_)*/V*	*v_av_*	*v_max_*	*v*_3*d.av*_	*v_max_/v_av_*	*v*_3*d.av*_ */v_av_*	*b*_1_	*b*_0.3*d*_	*b*_0_	*b*_0,*Set*_	*b*_0.3*d*_ */b*_1_	*b*_0_ */b*_1_	*b*_0,*Set*_ */b*_1_
(cm)	(-)	(-)	(kN)	(kN)	(%)	(kN/m)	(kN/m)	(kN/m)	(-)	(-)	(m)	(m)	(m)	(m)	(%)	(%)	(%)
200	10	0.25	1299	551	42.4	123.6	139.0	112.2	1.12	0.91	10.51	4.91	9.35	11.59	46.7	89.0	110.2
200	10	0.5	1310	601	45.9	124.6	129.7	122.3	1.04	0.98	10.51	4.91	10.10	10.71	46.7	96.0	101.8
200	10	0.75	1310	658	50.2	124.6	140.4	133.8	1.13	1.07	10.51	4.91	9.33	9.79	46.7	88.8	93.1
200	10	1	1314	711	54.1	125.0	155.9	144.7	1.25	1.16	10.51	4.91	8.43	9.08	46.7	80.2	86.4
200	10	1.5	1330	792	59.5	126.6	177.5	161.2	1.40	1.27	10.51	4.91	7.50	8.25	46.7	71.3	78.5
200	10	2	1348	843	62.5	128.2	190.1	171.6	1.48	1.34	10.51	4.91	7.09	7.86	46.7	67.5	74.7

**Table A9:** Analysis of the shear force distribution in the control perimeter 0.5*d* with 240 cm shear cap size.

c1 = c2	c/d	α1	*V*	*V* (*b*_0.3*d*_)	*V* (*b*_0.3*d*_)*/V*	*v_av_*	*v_max_*	*v*_3*d.av*_	*v_max_/v_av_*	*v*_3*d.av*_ */v_av_*	*b*_1_	*b*_0.3*d*_	*b*_0_	*b*_0,*Set*_	*b*_0.3*d*_ */b*_1_	*b*_0_ */b*_1_	*b*_0,*Set*_ */b*_1_
(cm)	(-)	(-)	(kN)	(kN)	(%)	(kN/m)	(kN/m)	(kN/m)	(-)	(-)	(m)	(m)	(m)	(m)	(%)	(%)	(%)
240	12	0.25	1343	342	25.5	131.4	149.5	113.1	1.14	0.86	10.23	3.03	8.98	11.88	29.6	87.8	116.2
240	12	0.5	1380	426	30.8	134.9	167.5	140.6	1.24	1.04	10.23	3.03	8.24	9.82	29.6	80.6	96.0
240	12	0.75	1395	530	38.0	136.4	244.5	175.1	1.79	1.28	10.23	3.03	5.71	7.97	29.6	55.8	77.9
240	12	1	1398	631	45.1	136.7	314.8	208.4	2.30	1.52	10.23	3.03	4.44	6.71	29.6	43.4	65.6
240	12	1.5	1396	792	56.7	136.5	417.2	261.7	3.06	1.92	10.23	3.03	3.35	5.34	29.6	32.7	52.2
240	12	2	1405	901	64.1	137.4	479.3	297.6	3.49	2.17	10.23	3.03	2.93	4.72	29.6	28.7	46.2

**Table A10:** Analysis of the shear force distribution in the control perimeter 2*d* with 240 cm shear cap size.

c1 = c2	c/d	α1	*V*	*V* (*b*_0.3*d*_)	*V* (*b*_0.3*d*_)/*V*	*v_av_*	*v_max_*	*v*_3*d.av*_	*v_max_/v_av_*	*v*_3*d.av*_ */v_av_*	*b*_1_	*b*_0.3*d*_	*b*_0_	*b*_0,*Set*_	*b*_0.3*d*_ */b*_1_	*b*_0_/*b*_1_	*b*_0,*Set*_/*b*_1_
(cm)	(-)	(-)	(kN)	(kN)	(%)	(kN/m)	(kN/m)	(kN/m)	(-)	(-)	(m)	(m)	(m)	(m)	(%)	(%)	(%)
240	12	0.25	1280	455	35.6	105.7	120.4	92.7	1.14	0.88	12.11	4.91	10.63	13.81	40.6	87.7	114.0
240	12	0.5	1298	506	39.0	107.2	114.3	102.9	1.07	0.96	12.11	4.91	11.36	12.61	40.6	93.8	104.1
240	12	0.75	1302	565	43.4	107.5	120.3	115.0	1.12	1.07	12.11	4.91	10.83	11.33	40.6	89.4	93.5
240	12	1	1308	622	47.5	108.0	136.1	126.6	1.26	1.17	12.11	4.91	9.61	10.33	40.6	79.3	85.3
240	12	1.5	1328	710	53.5	109.6	158.8	144.6	1.45	1.32	12.11	4.91	8.36	9.19	40.6	69.0	75.8
240	12	2	1349	767	56.8	111.4	172.2	156.0	1.55	1.40	12.11	4.91	7.83	8.65	40.6	64.7	71.4

Graphical results of numerical analysis of the shear forces in the control parameter depending on the shear cap height and shear cap width are presented in Figure A1, Figure A2, Figure A3, Figure A4 and Figure A5.
